# Peripheral inflammatory markers and clinical correlations in patients with frontotemporal lobar degeneration with and without the *C9orf72* repeat expansion

**DOI:** 10.1007/s00415-019-09552-1

**Published:** 2019-09-26

**Authors:** Kasper Katisko, Eino Solje, Paula Korhonen, Olli Jääskeläinen, Sanna Loppi, Päivi Hartikainen, Anne M. Koivisto, Aleksi Kontkanen, Ville E. Korhonen, Seppo Helisalmi, Tarja Malm, Sanna-Kaisa Herukka, Anne M. Remes, Annakaisa Haapasalo

**Affiliations:** 1 Institute of Clinical Medicine-Neurology, University of Eastern Finland, Kuopio, Finland; 2 MRC Oulu, Oulu University Hospital, Oulu, Finland; 3 Research Unit of Clinical Neuroscience, Neurology, University of Oulu, Oulu, Finland; 4 Neuro Center, Neurology, Kuopio University Hospital, Kuopio, Finland; 5 Neuro Center, Neurosurgery, Kuopio University Hospital, 70029 Kuopio, Finland; 6 A.I. Virtanen Institute for Molecular Sciences, University of Eastern Finland, Neulaniementie 2, 70211 Kuopio, Finland

**Keywords:** Frontotemporal lobar degeneration, Frontotemporal dementia, Parkinsonism, Disease progression, *C9orf72*, Inflammation, Cytokines

## Abstract

In this study, our aim was to evaluate potential peripheral inflammatory changes in frontotemporal lobar degeneration (FTLD) patients carrying or not the *C9orf72* repeat expansion. To this end, levels of several inflammatory markers (MCP-1, RANTES, IL-10, IL-17A, IL-12p, IFN-γ, IL-1β, IL-8, and hs-CRP) and blood cells counts in plasma and/or serum of FTLD patients (*N* = 98) with or without the *C9orf72* repeat expansion were analyzed. In addition, we evaluated whether the analyzed peripheral inflammatory markers correlated with disease progression or distinct clinical phenotypes under the heterogenous FTLD spectrum. Elevated levels of pro-inflammatory RANTES or MCP-1 and decreased levels of anti-inflammatory IL-10 were found to associate with Parkinsonism and a more rapid disease progression, indicated by longitudinal measurements of either MMSE or ADCS-ADL decline. These findings were observed in the total cohort in general, whereas the *C9orf72* repeat expansion carriers showed only slight differences in IL-10 and hemoglobin levels compared to non-carriers. Furthermore, these *C9orf72* repeat expansion-associated differences were observed mostly in male subjects. The females in general showed elevated levels of several pro-inflammatory markers compared to males regardless of the *C9orf72* genotype. Our study suggests that pro-inflammatory changes observed in the early symptomatic phase of FTLD are associated with distinct clinical profiles and a more rapid disease progression, and that the *C9orf72* repeat expansion and gender may also affect the inflammatory profile in FTLD.

**Electronic supplementary material:**

The online version of this article (10.1007/s00415-019-09552-1) contains supplementary material, which is available to authorized users.

## Introduction

Accumulating data have implicated that immunological alterations may play a role in the pathogenesis of both genetic and sporadic frontotemporal lobar degeneration (FTLD) [1–6]. Especially the most common genetic cause of FTLD, the *C9orf72* hexanucleotide repeat expansion (HRE) [7, 8], has been associated with immune system regulation [1–3]. Loss of function of the *C9orf72* gene in murine models recapitulates human autoimmune disease with severe autoimmune phenotypes, high mortality rate, and increased levels of inflammatory cytokines, such as IFN-γ, IL-17A, IL-12p, IL-10, IL-1β, and MCP-1, as well as alterations in hemoglobin levels and peripheral blood cell counts (leukocytes and thrombocytes). The inflammatory phenotypes were substantially more severe in total *C9orf72* knock-out (−/−) mouse models, but heterozygous knock-out mice (+/−), modeling haploinsufficiency due to the *C9orf72* HRE in human FTLD patients, also showed mild inflammatory alterations [1–3]. Moreover, studies in FTLD patients in general have indicated genetic association to immunoregulatory HLA regions [4, 5], association with autoimmune conditions [9–12], and, on the other hand, decreased prevalence of cancer [13], all indicating potential systemic immune system alterations in FTLD.

In this study, we have evaluated systemic inflammatory changes in FTLD patients carrying or not the *C9orf72* HRE by analyzing plasma/serum levels of several inflammatory markers and peripheral blood cell counts. In addition, we evaluated the association between these inflammatory markers and clinical features or disease progression of the patients. We observed that distinct inflammatory alterations associated with both disease progression and clinical manifestations in patients with FTLD.

## Methods

### Study population

A total of 98 FTLD patients were included in this study. Of these, 71 patients had bvFTD, 21 had PPA (17 nfvPPA, 4 svPPA) and 6 had FTLD-motoneuron disease (FTLD-MND) (Table 1). Patients were diagnosed in Kuopio University Hospital during the years 1998–2016 by a neurologist specialized in memory diseases. Patients were diagnosed with at least probable FTLD according to the latest diagnostic criteria by Rascovsky et al. for bvFTD and by Gorno-Tempini et al. for PPA [14, 15]. Patients with FTLD-MND had at least probable bvFTD or PPA, and a clinically clear manifestation of motoneuron disease. Patients that were diagnosed before 2011 (*N* = 48) were originally diagnosed according to the Neary criteria [16] and retrospectively confirmed to meet the latest (2011) diagnostic criteria. Of the 98 FTLD patients, 27 had a definite, and 71 a probable diagnosis of FTLD according to the latest criteria [14, 15].

**Table 1:** Clinical characteristics of FTLD patients included in the study

	FTLD total *N* (%)^c^	FTLD with *C9orf72* HRE *N* (%)^c^	FTLD without *C9orf72* HRE *N* (%)^c^
Total number (*N*)	98^a^	27^a^	58^a^
CBA analysis (plasma)	50 (51%)	22 (81%)	28 (48%)
Simoa (sera)	91 (93%)	26 (96%)	52 (90%)
hs-CRP	44 (45%)	19 (70%)	24 (41%)
Gender (F/M, %)	53/47%	52/48%	57/43%
Mean age^b^ ± SD	64.7 ± 8.8	61.2 ± 9.3	66.2 ± 8.0
bvFTD (*N*)	71 (72%)	20 (74%)	39 (67%)
PPA (*N*)	21 (21%)	5 (19%)	15 (26%)
nfvPPA (*N*)	17 (17%)	5 (19%)	11 (19%)
svPPA (*N*)	4 (4%)	0 (0%)	4 (7%)
FTLD-MND (*N*)	6 (6%)	2 (7%)	4 (7%)
FTLD with Parkinsonism (*N*)^d^	23 (23%)	8 (30%)	13 (22%)
FTLD with psychotic symptoms (*N*)^d^	34 (35%)	10 (37%)	18 (31%)

^a^FTLD total group includes overall 13 patients without known *C9orf72* HRE status

^b^Age is calculated from the date of the blood sample

^c^Percentages are calculated from the total number of cases in each column

^d^Out of the FTLD patients with Parkinsonism (*N* = 23), 17 patients had Parkinsonism symptoms at the time of the blood sample and FTLD diagnosis and 6 patients developed Parkinsonism after the blood sample and FTLD diagnosis. All of the patients with psychotic symptoms (*N* = 34) had their first psychotic symptoms before the blood sample and FTLD diagnosis

### Profiling the clinical features and disease progression

To evaluate the correlation of inflammatory markers to distinct clinical manifestations, data for the presence or absence of several FTLD-related clinical features (psychotic symptoms, Parkinsonism and motoneuron symptoms) were collected as dichotomous variables. The inclusion criteria for each of these clinical features are described in Online Resource Supplementary File 1.

The rate of disease progression was measured with two questionnaires, the Mini Mental State Examination (MMSE, 0–30p) for cognitive decline and Activities of Daily Living scale (ADCS-ADL, 0–78p) for functional decline. Decline rates were measured for patients that had data from at least two time points, and the variable was calculated as decline in points per months (higher score indicating more rapid decline). Data for ADCS-ADL decline were available from 22 FTLD patients and data for MMSE decline were available from 31 patients. The follow-up period between tests varied between 6 and 33 months (mean 13.7, median 11 months) in ADCS-ADL questionnaire and between 3 and 78 months (mean 22.6, median 12 months) in MMSE test.

### Genetic analyses

Repeat-primed polymerase chain reaction assay (RP-PCR) [8] and amplicon length analysis [17] were used to confirm the presence or absence of the *C9orf72* HRE. Out of the 98 FTLD subjects, 58 patients were confirmed as *C9orf72* HRE non-carriers (*N* = 58) (< 30 repeats) and 27 patients carried the HRE (> 30 repeats). The *C9orf72* HRE was present in 20 bvFTD patients, five nfvPPA patients and two FTLD-MND patients (there were no *C9orf72* HRE carriers presenting with the svPPA phenotype). The *C9orf72* HRE genotyping data were not available for 13 FTLD patients, and these patients were included only to the analyses comparing the inflammatory marker levels with disease phenotypes/progression in the whole cohort. We did not systematically screen other FTLD mutations such as *MAPT*, *GRN* and *CHMP2B* mutations, as our previous studies (partly including the same patients as this present one) have shown that these mutations are extremely rare in the Finnish population [18–20].

### Peripheral blood cell count (hemoglobin, thrombocytes, leukocytes) and high-sensitive C-reactive protein analyses (plasma CRP)

Peripheral blood cell count was analyzed as part of the routine diagnostic laboratory tests made in the memory outpatient clinic in Kuopio University Hospital. If blood cell counts were measured more than once (range 1–3) during the diagnostic phase, median value from these tests was used for each variable. Samples taken during an acute infection were excluded from the calculations. After exclusions, hemoglobin levels were available for 87 patients, and thrombocyte and leukocyte levels for 82 patients. High-sensitive C-reactive-protein (hs-CRP) levels were analyzed from FTLD patients’ plasma samples (*N* = 44) with Cobas 6000 (c 501)—analyzer, Hitachi High Technology Co, Tokyo, Japan (Online Resource Supplementary File 1).

### Cytokine and chemokine analyses in patients with FTLD

Selected cytokines/chemokines (MCP-1, RANTES, IL-10, IL-17A, IL-12p and IFN-γ) from plasma of selected patients (*N* = 50) were first analyzed with Cytometric Bead Array (CBA), a flow cytometry-based application allowing simultaneous quantification of multiple proteins. Only the levels of MCP-1 and RANTES were detectable using this method. The levels of MCP-1 and IL-10 were also analyzed from 91 patients with the ultra-sensitive Single Molecule Array (Simoa) technology [21], which enables detection of molecules in extremely low (femtomolar) concentrations. RANTES immunoassay was not available for Simoa at the time of the study. In addition, proinflammatory IL-8 and IL-1β were included in the Simoa analyses (*N* = 91). Details of the CBA and Simoa analyses are described in Online Resource Supplementary File 1. The measured cytokines were selected based on the reported alterations in the *C9orf72* knock-out murine model studies [1–3]. Additionally, RANTES and IL-8 were chosen based on assay availability and based on previous studies showing alterations in the peripheral levels of these cytokines especially in ALS and Parkinson’s disease [22–25].

### Statistical analyses

**Fig. 1:**
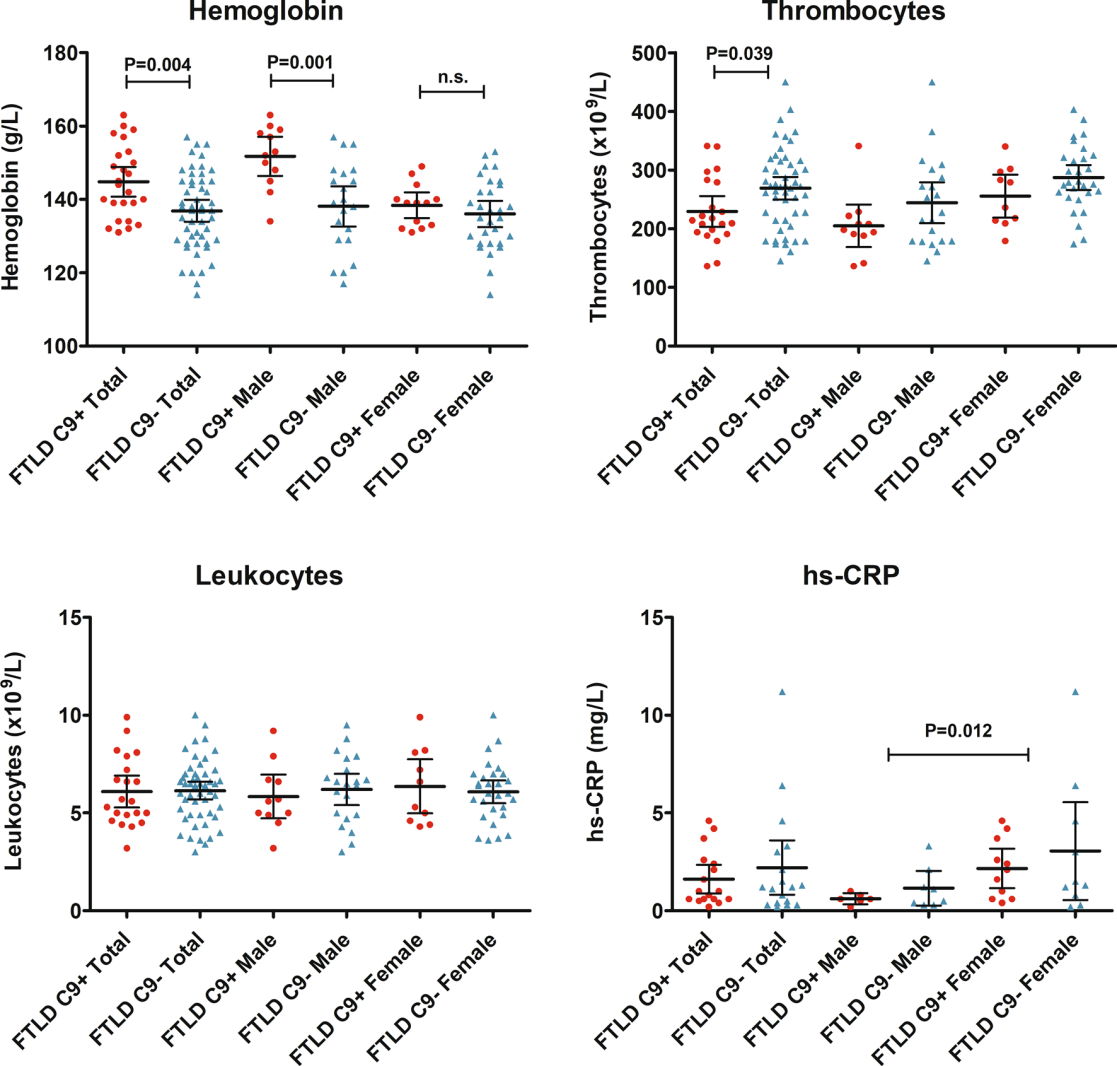
Peripheral blood cell counts and plasma hs-CRP concentrations in FTLD patients with or without the *C9orf72* HRE. Symbols represent separate cases, and horizontal lines represent median with interquartile range. Mann–Whitney *U* test was used to compare groups separately. For hs-CRP, the significant *p* value is calculated for all males compared to all females, regardless of the *C9orf72* HRE status (comparison between genders)

**Fig. 2:**
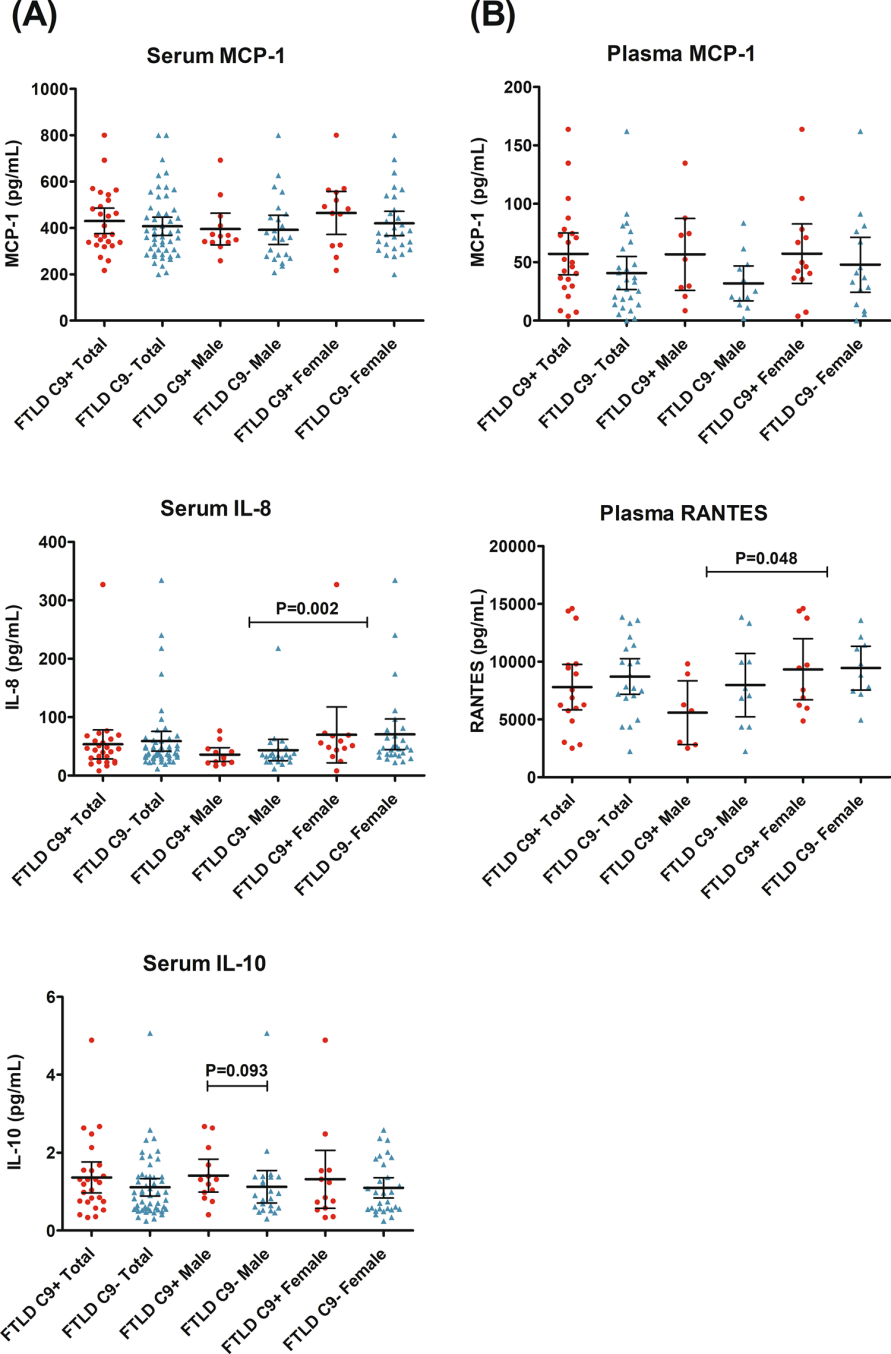
**a** Concentrations of MCP-1, IL-8 and IL-10 in serum of FTLD patients carrying or not the *C9orf72* HRE measured using single molecule array (Simoa). The data for IL-1β is not shown due to extremely low concentrations observed in most cases. **b** Plasma MCP-1 and RANTES, measured using cytometric bead array (CBA). **a**, **b** Symbols represent separate cases, and horizontal lines represent median with interquartile range. Mann–Whitney *U* test was used to compare groups separately. For IL-8 and RANTES, the significant *p* value is calculated for all males compared to all females, regardless of the *C9orf72* HRE status (comparison between genders). For IL-10, the difference between male *C9orf72* HRE carriers and non-carriers was significant when outliers were excluded (*p* = 0.049)

**Fig. 3:**
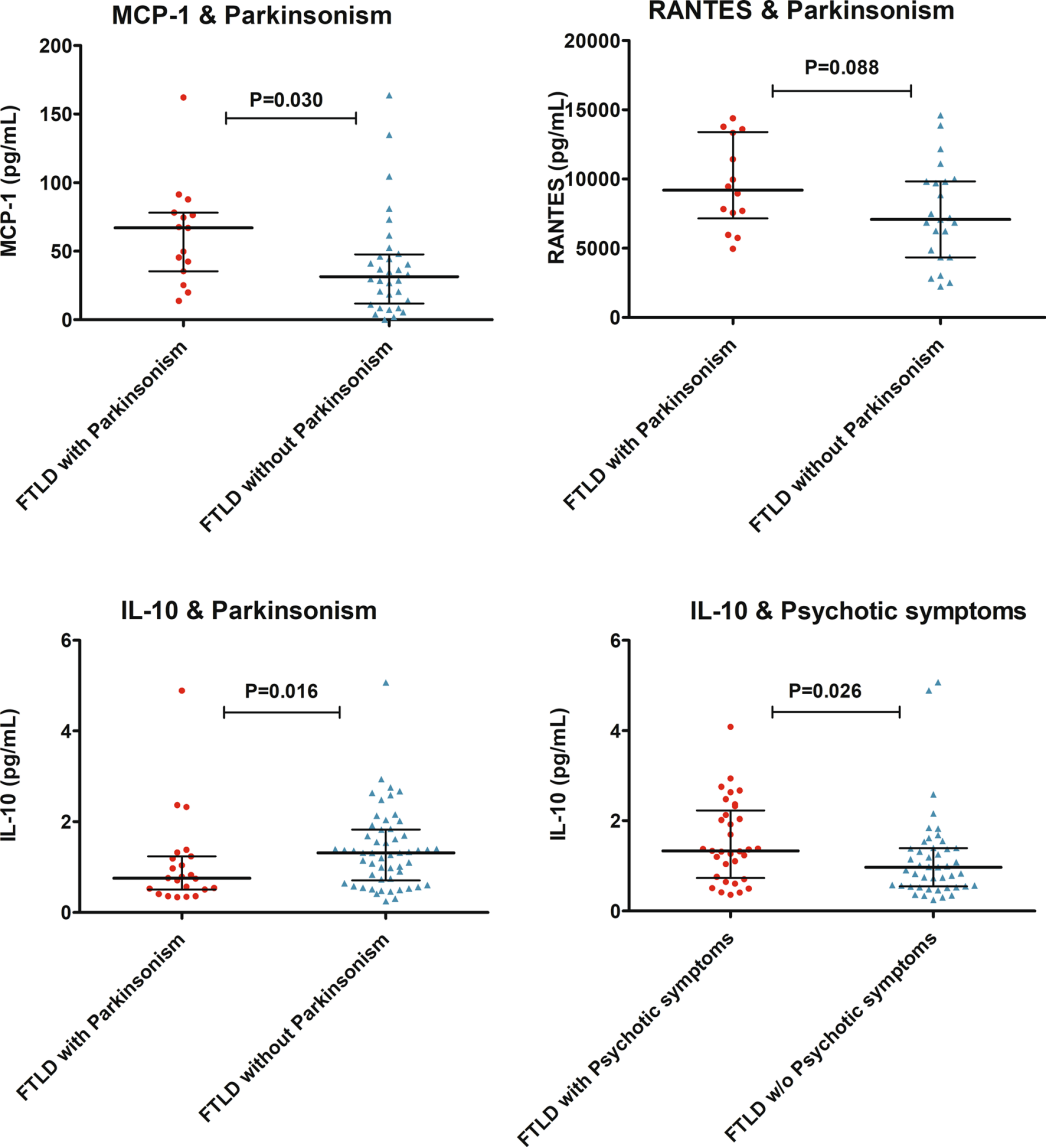
Concentrations of the inflammatory cytokines MCP-1 (plasma), RANTES (plasma) and IL-10 (serum) in FTLD patients with or without Parkinsonism or psychotic symptoms. Symbols represent separate cases, and horizontal lines represent median with interquartile range. Mann–Whitney *U* test was used to compare the groups

All statistical analyses were performed using IBM SPSS Statistics 25. The Shapiro–Wilk test was used to test the normality of distribution of the data. Due to the non-normal distribution, high skewness and several outliers non-parametric tests were systematically used for the cytokines, hs-CRP and peripheral blood count factors when comparing different groups; Mann–Whitney *U* test was used for continuous variables to compare two independent groups and Spearman’s rank correlation test was used to analyze correlations between continuous variables. The effect of age and gender to the analyzed markers and clinical variables were analyzed separately to evaluate the role of these factors as potential confounders. If one or more of the variables (inflammatory marker or clinical feature) was significantly affected by age or gender status, analysis of covariance (ANCOVA) or multiple linear regression was performed with age and/or gender as covariate(s) (gender as a fixed factor and age as a covariate in ANCOVA). For the covariance analysis, non-normally distributed data were transformed with natural logarithm transformation to enable parametric testing. Data for the measured markers in Table 2 and in Figs. 1, 2 and 3 are presented as median with interquartile range. Chi-square test was used to compare two categorical variables. *p* value of ≤ 0.05 was considered as statistically significant. Figures were created with GraphPad Prism 5 software.

**Table 2:** Inflammatory molecule concentrations and peripheral blood cell counts in FTLD patients with and without the *C9orf72* HRE

Molecules	FTLD with *C9orf72* HRE			FTLD without *C9orf72* HRE			*p* value
	Males	Females	Males and females	Males	Females	Males and females	
Plasma MCP-1 (pg/ml)	52.51 (56.58)	46.12 (38.68)	47.98 (46.09)	25.26 (38.71)	36.74 (62.38)	34.05 (50.96)	NS
Plasma RANTES (pg/ml)	5758.24 (6141.67)	8508.65 (7768.83)	6878.30 (4453.53)	7400.79 (6469.65)	9343.94 (4195.54)	8349.42 (4433.47)	NS
Serum MCP-1 (pg/ml)	364.57 (92.42)	481.91 (233.92)	390.43 (190.43)	359.51 (183.52)	378.80 (186.89)	377.60 (169.89)	NS
Serum IL-10 (pg/ml)	1.31 (1.00)	0.85 (0.99)	1.21 (0.84)	0.86 (0.80)	0.97 (1.22)	0.90 (0.83)	NS*
Serum IL-8 (pg/ml)	32.89 (31.84)	51.28 (30.52)	44.69 (39.76)	34.76 (22.35)	47.23 (36.90)	39.28 (28.24)	NS
Serum IL-1β (pg/ml)	1.52 (2.93)	0.30 (NA)	0.37 (1.38)	0.71 (3.25)	0.50 (6.35)	0.51 (4.23)	NS
hs-CRP (mg/l)	0.60 (0.43)	2.10 (3.10)	1.00 (1.90)	0.80 (1.55)	1.40 (4.38)	1.20 (2.70)	NS
Leukocytes (×10^9^/l)	5.60 (1.80)	5.95 (3.58)	5.60 (2.80)	6.50 (2.70)	6.00 (1.95)	6.40 (2.10)	NS
Thrombocytes (×10^9^/l)	198 (34.00)	258 (85.50)	214^1^ (88.50)	228 (115.00)	279 (66.25)	272^1^ (101.25)	0.039^1^
Hemoglobin (g/l)	153^1^ (13.0)	139 (9.5)	144^2^ (16.0)	139^1^ (18.5)	136 (16.3)	137^2^ (16.0)	0.001^1^ 0.004^2^

Concentrations are presented as median (interquartile range, IQR). Plasma MCP-1 and RANTES were analyzed with cytometric bead array, and serum MCP-1, IL-10, IL-8 and IL-1β with single molecule array. Mann–Whitney *U* test was used to compare groups separately (three comparisons per row: between all *C9orf72* HRE carriers and non-carriers, between male carriers and male non-carriers, and between female carriers and female non-carriers). Comparisons between genders are presented in Figs. 1 and 2

*p* value column presents statistically significant differences in each row, and the superscript numbers indicate which two groups were compared

*NS* indicates no significant differences in any of the three separate pairwise comparisons per row

*In IL-10 levels, *C9orf72* HRE carrier males showed a trend for higher IL-10 concentration compared to non-carrier males (*p* = 0.093), being significant when outliers were excluded (*p* = 0.049)

## Results

Here, we have assessed whether potential peripheral inflammatory changes associate with clinical features or disease progression of FTLD. We compared the levels of different blood-based inflammatory markers between FTLD patients carrying or not the *C9orf72* HRE and performed correlation analyses with distinct clinical features of FTLD. Characteristics of the FTLD cohort are presented in Table 1.

First, blood parameters including blood cell counts and hs-CRP were investigated. The *C9orf72* HRE carriers were observed to have higher levels of hemoglobin compared to non-carriers (*U* = 895, *p* = 0.004). The difference remained statistically significant also after ANCOVA with age and gender as covariates (*p* = 0.002). However, the finding was observed mostly in males, as when compared separately, *C9orf72* HRE carrier males had higher hemoglobin levels compared to non-carrier males (*U* = 209, *p* = 0.001). The difference in females was not significant. Moreover, the *C9orf72* HRE carriers had lower levels of thrombocytes compared to non-carriers (*U* = 361, *p* = 0.039), but the difference was no longer significant after adjusting for age and gender (*p* = 0.06). There were no differences between the *C9orf72* HRE carriers and non-carriers in the total leukocyte counts or hs-CRP concentrations. Overall, levels of hs-CRP were higher in females compared to males (*U* = 73, *p* = 0.012) regardless of whether the patients carried or not the *C9orf72* HRE. In addition, levels of hemoglobin were lower (*U* = 957, *p* = 0.010), and levels of thrombocytes higher (*U* = 336, *p* = 0.001) in females compared to males (Table 2, Fig. 1).

To assess inflammation-related blood-based cytokines and chemokines, the levels of MCP-1, RANTES, IL-10, IL-17A, IL-12p, IFN-γ were first measured in plasma using CBA analysis. We found that only MCP-1 and RANTES levels were detectable with this method. There were no differences in MCP-1 or RANTES plasma levels between *C9orf72* HRE carriers and non-carriers. In general, the females had higher levels of RANTES compared to males regardless of whether they carried or not the *C9orf72* HRE (*U* = 105, *p* = 0.048) (Table 2, Fig. 2).

Next, the levels of MCP-1, IL-10, IL-8, and IL-1β were measured with the ultrasensitive Simoa method in patient sera. The levels of IL-1β were below the quantification range in some FTLD patients and 61.5% (56/91) of the patients had to be excluded from the analyses. There were no detectable differences between the *C9orf72* HRE carriers and non-carriers in the serum levels of IL-8, MCP-1, or IL-1β. The *C9orf72* HRE carriers showed a non-significant trend towards higher levels of IL-10 compared to non-carriers in male subjects (*U* = 201, *p* = 0.093). When obvious outliers were excluded from the data (one male outlier in the non-carrier group and one female outlier in the carrier group), the difference was significant in male subjects (*C9orf72* HRE carriers vs. non-carriers, *U* = 201 *p* = 0.049). When both genders were included in the analysis, IL-10 levels did not differ significantly between *C9orf72* HRE carriers and non-carriers (*U* = 753, *p *= 0.202). Overall, females had higher levels of IL-8 regardless of whether they carried or not the *C9orf72* HRE (*U* = 427, *p* = 0.002) (Table 2, Fig. 2).

Finally, we examined whether the peripheral inflammatory markers correlated with clinical features of FTLD patients. These analyses revealed that only IL-8 levels correlated with age (*r*_s_ = 0.231, *p* = 0.029). The IL-8 analyses were thus also corrected for age in addition to gender in ANCOVA, but there were no statistically significant differences in IL-8 levels between the *C9orf72* HRE genotypes or clinical subgroups (such as FTLD Parkinsonism vs. non-Parkinsonism). A history of an autoimmune disease was detected in 11 patients, but the measured inflammatory marker levels did not differ in these patients compared to those without autoimmune disease. Three patients had systemic (oral administration) immunomodulatory medication but showed no distinct differences in the marker levels compared to the other patients.

FTLD patients with Parkinsonism showed significantly higher levels of plasma MCP-1 (*U* = 345, *p* = 0.030) and, conversely, lower levels of the anti-inflammatory IL-10 (*U* = 404, *p* = 0.016) compared to FTLD patients without Parkinsonism. Furthermore, the patients with Parkinsonism showed a non-significant trend towards higher levels of RANTES (*U* = 216, *p* = 0.088). As RANTES levels were significantly affected by gender, the RANTES levels in FTLD with Parkinsonism vs. FTLD without Parkinsonism were further compared using ANCOVA with gender being as covariate. In the covariance analysis, the difference remained as a non-significant trend (*p* = 0.059). Additionally, patients displaying psychotic symptoms had elevated levels of IL-10 (*U* = 963, *p* = 0.026) (Fig. 3). There were no differences in age or gender distribution between the Parkinsonism vs. non-Parkinsonism groups or between the psychotic vs. non-psychotic groups. No differences in any of the measured cytokines or chemokines were observed when comparing patients with or without motoneuron symptoms (notably only seven patients showed motoneuron symptoms).

**Fig. 4:**
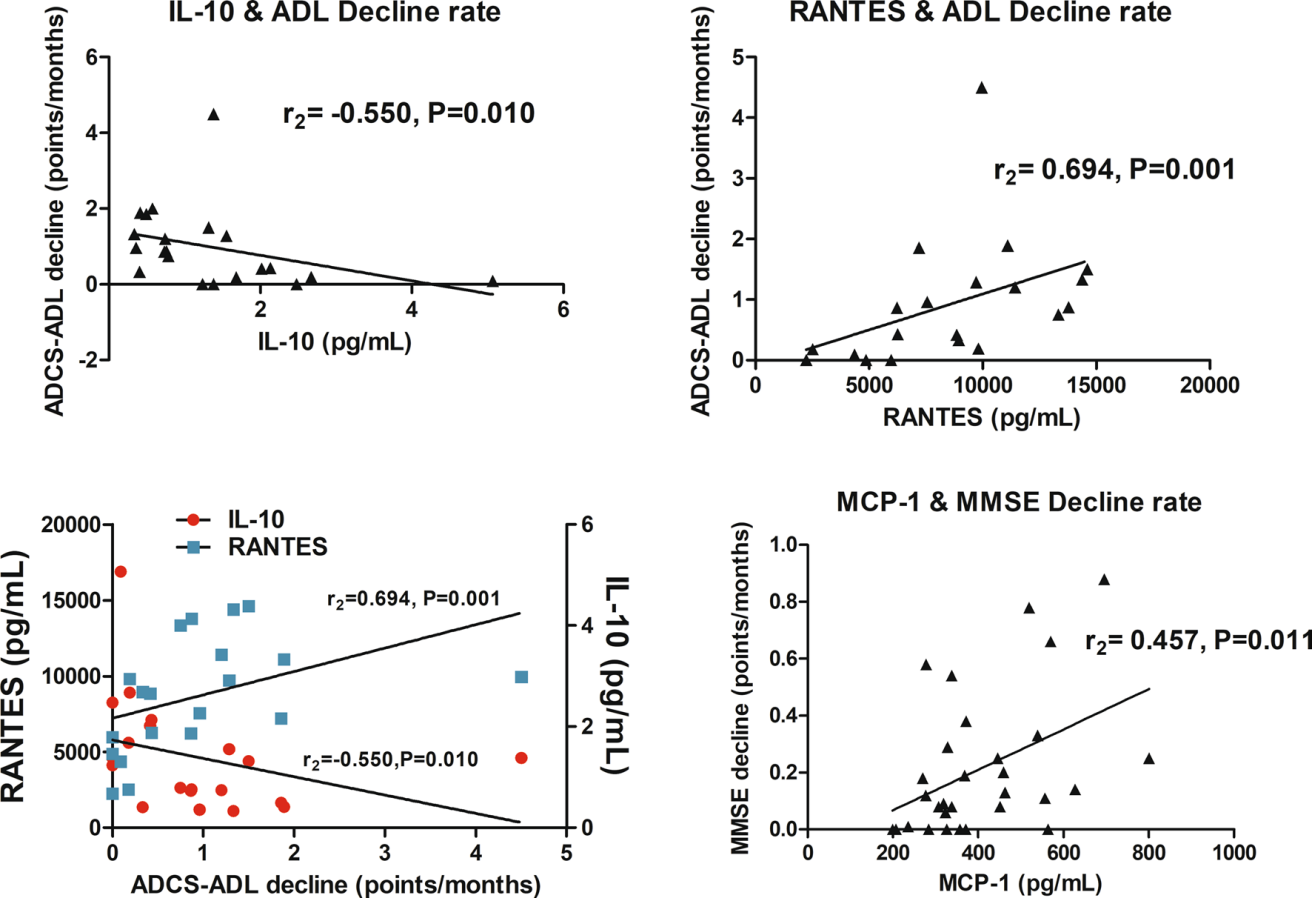
Correlations of the levels of inflammatory cytokines (plasma RANTES, serum MCP-1 and serum IL-10) and functional or cognitive decline rates. Functional decline was assessed using ADCS-ADL and cognitive decline using MMSE examination. Higher decline ratio score indicates more rapid progression. Correlation analyses were performed with Spearman’s rank correlation test

We then investigated whether the measured cytokine or chemokine levels associated with cognitive or functional decline using MMSE and ADCS-ADL, respectively. None of the cytokines showed correlation with baseline MMSE or ADCS-ADL scores. However, the longitudinal functional decline rate measured in ADCS-ADL (points per months, higher score indicating more rapid decline) showed a strong positive correlation to plasma levels of RANTES (*r*_s_ = 0.694, *p* = 0.001, which remained significant also after adjusting for age and gender; *p* = 0.023), and a moderate negative correlation to serum levels of the anti-inflammatory IL-10 (*r*_s_ = − 0.550, *p* = 0.010). The longitudinal cognitive decline rate measured with MMSE showed a moderate positive correlation to serum levels of MCP-1 (*r*_s_ = 0.457, *p* = 0.011) (Fig. 4). Age at the time of the blood sampling or gender did not correlate with baseline or longitudinal MMSE or ADCS-ADL scores, indicating that neither age nor gender affected the observed results. Only plasma MCP-1 showed a weak correlation (*r*_s_ = 0.391, *p* = 0.010) with the duration of the time from acquiring the blood sample to the analysis, indicating that the variation in storage times did not significantly affect our results. Correlation analyses between the different cytokines indicated that only IL-8 showed a moderate positive correlation to serum MCP-1 (*r*_s_ = 0.553, *p* < 0.001) and a weak positive correlation to IL-10 (*r*_s_ = 0.239, *p* = 0.024) levels. Serum MCP-1 levels correlated with MCP-1 levels in plasma (*r*_s_ = 0.310, *p* = 0.043), cross-validating the result.

## Discussion

Increasing evidence suggests that immune system alterations, including inflammation and autoimmunity, are associated with both sporadic and genetic forms of FTLD [4, 10, 12, 26, 27]. Several murine model studies have indicated that *C9orf72* loss-of-function leads to severely altered peripheral inflammatory marker levels (cytokines, autoantibodies and peripheral blood counts) coinciding with autoimmune disease-like phenotypes and increased mortality [1–3]. To our knowledge, the present study is the first one describing systemic inflammatory markers in human patients carrying the *C9orf72* HRE. It should be noted that only a few studies have so far addressed the levels of peripheral inflammatory molecules in FTLD in general [28–30]. Elevated levels of IL-6 in sporadic FTLD and FTLD patients with *GRN* mutations have been observed [29, 30]. In another study, serum and CSF levels of 27 inflammatory molecules (including IL-6) were screened, but no differences in the serum in *GRN* mutation-carrying or sporadic FTLD patients were identified [28]. We observed that out of the measured factors (MCP-1, RANTES, IL-10, IL-17A, IL-12p, IFN-γ, IL-8, IL-1β, hemoglobin, thrombocytes, leukocytes and hs-CRP), only hemoglobin and IL-10 differed between *C9orf72* HRE carriers and non-carriers (higher levels in the carriers). These differences were observed mostly in male subjects, suggesting a possible gender-specific effect of the *C9orf72* HRE. However, the reason and underlying mechanisms of the elevated levels of IL-10 and hemoglobin observed here in *C9orf72* HRE-carrying males require further studies. In contrast to our data in male patients, *C9orf72* homozygous knock-out mice previously showed decreased levels of hemoglobin [1, 2]. Thus, in patients harboring one healthy and one expanded allele, the *C9orf72* HRE-related haploinsufficiency does not appear to alter peripheral cytokines or blood count factors in a similar manner to the total knock-out mice. In the present study, we also observed several other gender-specific differences, as the levels of IL-8, RANTES and hs-CRP were all higher in females compared to males, regardless of whether the patients carried the *C9orf72* HRE or not. Although these differences might not be specifically related to FTLD, they suggest that the gender may potentially influence the immunological profiles in FTLD.

Associations between peripheral inflammatory molecules and cognitive or psychometric measures or between peripheral inflammation and longitudinal data reflecting disease progression in FTLD patients have not been so far reported [28–30]. Our present data provide novel evidence for the association between specific altered inflammatory markers and disease progression or clinical manifestation of FTLD. The levels of IL-10 correlated negatively and those of RANTES positively to a more rapid disease progression (measured with a decline rate in the ADCS-ADL score). Additionally, increased levels of MCP-1 correlated with a more rapid cognitive decline in FTLD patients as measured with a decline rate in MMSE. In line with these findings, higher plasma MCP-1 levels were also recently associated with a more rapid cognitive decline in Alzheimer’s disease patients [31]. Interestingly, MCP-1 and RANTES are pro-inflammatory immune mediators that recruit immune cells to the site of inflammation [32–34], whereas IL-10 is a key anti-inflammatory cytokine [35, 36]. Our results suggest that high levels of IL-10 might be protective against rapid disease progression and, conversely, low levels of IL-10 may associate with a more rapid progression in FTLD patients. Elevated levels of the pro-inflammatory RANTES and MCP-1, in turn, could lead to accelerated disease progression, even though it is also possible that inflammation represents a secondary phenomenon rather than a driver in patients with a more rapid progression.

Interestingly, patients manifesting with Parkinsonism showed lower levels of IL-10, elevated levels of MCP-1, and a nonsignificant trend towards elevated levels of RANTES, suggesting that the clinical FTLD phenotype with Parkinsonism is associated with a disadvantageous pro-inflammatory profile. This idea is supported by previous studies showing increased MCP-1 and RANTES levels in patients with Parkinson’s disease (PD) [24, 37], suggesting that increased levels of these cytokines may associate with extrapyramidal neuronal system decline. Moreover, alterations in cytokine levels, abnormal glial activation, and T-cell infiltration have been observed in PD patients, further pointing to inflammation as an important contributor to PD pathogenesis [38]. Another interesting finding in the present study was that FTLD patients with psychotic symptoms had elevated IL-10 levels. This finding is supported by previous reports showing that especially patients with first-episode schizophrenia or bipolar disorder with psychotic symptoms display elevated levels of IL-10 [39, 40]. Based on these observations, we hypothesize that inflammation associated with altered IL-10, MCP-1 and RANTES levels might at least partially underlie and predict the different clinical phenotypes or progression rates in patients under the heterogeneous FTLD spectrum. Moreover, the blood samples in this study were mainly collected at the early symptomatic phase of FTLD (at the first visit in the neurology clinic), which suggests that early pro-inflammatory cytokine changes may be disadvantageous.

The strengths of our study are that the frequency of the *C9orf72* HRE carriers in our cohort is considerably high and that the association between the *C9orf72* HRE carriers and inflammatory markers has not been previously studied. Our study provides novel information on the associations between inflammation and several carefully defined clinical features and disease progression. Furthermore, the Simoa used for IL-8, IL-10, IL-1β and MCP-1 analyses, provides ultrasensitive methodology that enables more reliable detection of low-grade inflammation compared to standard ELISA-based methods. The limitations include the fact that our cohort is rather limited in size for explicit conclusions and replication in other cohorts (from other geographical populations) is required to confirm our findings. As we did not include corrections for multiple comparisons, our results represent preliminary suggestions rather than explicit associations. Notably, as the observed differences related to disease progression and Parkinsonism were biologically rational (inverse effects of pro-inflammatory RANTES and MCP-1 and anti-inflammatory IL-10), the results are likely to suggest biological pathways rather than coincidental type 1 errors. Thus, validation of the present findings warrants further studies. We cannot exclude the fact that other factors (apart from age and gender), unrelated to the disease process, may have affected the results of this study. Additionally, as our study provides a cross-sectional analysis at one time point for the cytokine levels (early symptomatic phase of FTLD), further longitudinal assessments are needed in the future. Finally, we did not have the opportunity to include healthy controls in our study, and thus at this point cannot evaluate whether the observed differences are detected only inside the FTLD spectrum or whether the findings in specific subgroups (such as FTLD with Parkinsonism) would also differ from those in non-FTLD participants.

In conclusion, our study suggests that pro-inflammatory changes (indicated by elevated levels of the pro-inflammatory RANTES and MCP-1 and decreased levels of the anti-inflammatory IL-10) observed in the early symptomatic phase of FTLD are associated with distinct clinical profiles and a more rapid disease progression. Furthermore, we show that the *C9orf72* HRE and gender may affect the inflammatory profile in FTLD, although the observed differences related to the *C9orf72* HRE were not as prominent as in the previous studies in *C9orf72* knock-out murine models [1–3]. Future longitudinal studies in other cohorts are warranted to confirm the suggested correlation of the inflammatory profiles and clinical manifestations in FTLD in general and in FTLD patients carrying the *C9orf72* HRE.

## Electronic supplementary material

Below is the link to the electronic supplementary material.

Supplementary file1 (PDF 198 kb)
