# Supplementary material for: Peripheral inflammatory markers and clinical correlations in patients with frontotemporal lobar degeneration with and without the C9orf72 repeat expansion
Source: J Neurol. 2019 Sep 26;267(1):76–86. doi: 10.1007/s00415-019-09552-1 (PMC6954907; doi:10.1007/s00415-019-09552-1)
Supplement: Supplementary file 1 — Supplementary file1 (PDF 198 kb) [file 415_2019_9552_MOESM1_ESM.pdf]

Supplementary File 1 – Journal of Neurology

Peripheral Inflammatory Markers and Clinical Correlations in Patients with Frontotemporal Lobar Degeneration with and without the *C9orf72* Repeat Expansion

Kasper Katisko, Eino Solje, Paula Korhonen, Olli Jääskeläinen, Sanna Loppi, Päivi Hartikainen, Anne M Koivisto, Aleksi Kontkanen, Ville E Korhonen, Seppo Helisalmi, Tarja Malm, Sanna-Kaisa Herukka, Anne M Remes, Annakaisa Haapasalo

Corresponding author:

Annakaisa Haapasalo

PhD, Adjunct Professor, Research Director

A.I. Virtanen Institute, University of Eastern Finland

Neulaniementie 2, 70211, Kuopio, Finland

E-mail: annakaisa.haapasalo@uef.fi

Tel: +358403552768

Supplementary methods

Profiling the clinical features and disease progression

Data for presence or absence of several FTLD-related clinical features (psychotic symptoms, parkinsonism and motoneuron symptoms) was collected as dichotomous variables. To be included in the group of FTLD with psychotic symptoms, the patient must have had at least one evident psychotic symptom, i.e. either delusion or hallucination. FTLD with Parkinsonism was defined when at least two symptoms of the following were observed: resting tremor, bradykinesia, rigidity, prominent hypomimia, postural instability or loss of automatic movements. FTLD with motoneuron symptoms required at least "clinically possible" diagnosis according to the revised El Escorial criteria [1]. All of these clinical features were included if they were observed either at the disease onset or during the follow-up. The mean follow-up time was 33.8 months (range 3-123 months, median 24 months).

Cytometric Bead Array (CBA) analyses (plasma MCP-1, RANTES, IL-10, IL-17A, IL-12p, IFN-γ)

Cytokine concentrations were determined using BD CBA Flex Set system for human samples (BD Biosciences, San Jose, CA, USA). A total of 50 FTLD sample aliquots were thawed before the analysis. The samples were incubated for three hours with kit components (beads and detection reagent) according to manufacturer's instructions. After three washes with Wash Buffer, the samples were run with FACS Aria flow cytometer (BD Biosciences) and converted to Excel file by using FCAP Array 2.0.0 Software (Soft Flow Hungary Ltd, Pecs, Hungary). All analytes were quantified separately and the sample location on plate was randomized. All samples were analyzed as duplicates, and mean concentration calculated from the duplicates was used.

Single Molecule Array (Simoa) analyses (serum IL-1β, IL-8, IL-10 and MCP-1)

A total of 91 FTLD serum samples were thawed, divided in to four aliquots and frozen for future analysis. After a second thaw, the aliquots were mixed, centrifuged (10, 000 x *g* 5 min, + 22 ºC) and transferred to 96-well plates with calibrators and quality control samples. Serum IL-1β and IL-8 were quantified using Simoa HD-1 two-step Advantage digital immunoassays. The quantification of IL-10 and MCP-1 utilized three-step advantage digital immunoassays [2]. All analytes were quantified separately and the sample location on plate was randomized. All samples were analyzed as duplicates, and mean concentration calculated from the duplicates was used. Samples having a coefficient of variation higher than 15 % were excluded. The highest concentration of the assay`s quantification range was used for samples with both duplicate values exceeding the quantification range. Values below the quantification range were excluded (only IL-1β showed levels below the quantification range).

High-sensitive C-Reactive Protein analyses (plasma CRP)

A total of 44 plasma samples were thawed, divided into aliquots, and frozen before the analysis. After a second thaw, the aliquots were centrifuged and analyzed with particle enhanced immunoturbidimetric assay by Cobas 6000 (c 501) –analyzer, Hitachi High Technology Co, Tokyo, Japan. Measuring range was 0.15 – 20 mg/L. Samples with concentration below the measuring range (N=8) were considered as non-detectable and excluded from the analyses. One sample with a concentration of 28 mg/L (FTLD *C9orf72* HRE carrier) was excluded as such a high concentration likely reflects another inflammatory state, such as infection.

Supplementary references

1. Brooks BR, Miller RG, Swash M, Munsat TL (2000) El Escorial revisited: Revised criteria for the diagnosis of amyotrophic lateral sclerosis. Amyotroph Lateral Scler. https://doi.org/10.1080/146608200300079536
2. Rissin DM, Kan CW, Campbell TG, et al (2010) Single-molecule enzyme-linked immunosorbent assay detects serum proteins at subfemtomolar concentrations. Nat Biotechnol 28:595–599. https://doi.org/10.1038/nbt.1641
